# Inhibition of PFKFB3 induces cell death and synergistically enhances chemosensitivity in endometrial cancer

**DOI:** 10.1038/s41388-020-01621-4

**Published:** 2021-01-08

**Authors:** Yinan Xiao, Ling Jin, Chaolin Deng, Ye Guan, Eleftheria Kalogera, Upasana Ray, Prabhu Thirusangu, Julie Staub, Sayantani Sarkar Bhattacharya, Haotian Xu, Xiaoling Fang, Viji Shridhar

**Affiliations:** 1 Department of Experimental Pathology, Mayo Clinic, Rochester, MN USA; 2 Department of Obstetrics and Gynecology, the Second Xiangya Hospital, Central South University, Changsha, Hunan P.R. China; 3 Department of Gastroenterology and Hepatology, Mayo Clinic, Rochester, MN USA; 4 Department of Chemistry, University of Michigan, Ann Arbor, MI USA; 5 Division of Gynecologic Oncology, Mayo Clinic, Rochester, MN USA; 6 Department of Computer Science, Wayne State University, Detroit, MI USA

**Keywords:** Chemotherapy, Targeted therapies, Endometrial cancer, Apoptosis, Autophagy

## Abstract

The advanced or recurrent endometrial cancer (EC) has a poor prognosis because of chemoresistance. 6-Phosphofructo-2-kinase/fructose-2,6-bisphosphatase 3 (PFKFB3), a glycolytic enzyme, is overexpressed in a variety of human cancers and plays important roles in promoting tumor cell growth. Here, we showed that high expression of PFKFB3 in EC cell lines is associated with chemoresistance. Pharmacological inhibition of PFKFB3 with PFK158 and or genetic downregulation of PFKFB3 dramatically suppressed cell proliferation and enhanced the sensitivity of EC cells to carboplatin (CBPt) and cisplatin (Cis). Moreover, PFKFB3 inhibition resulted in reduced glucose uptake, ATP production, and lactate release. Notably, we found that PFK158 with CBPt or Cis exerted strong synergistic antitumor activity in chemoresistant EC cell lines, HEC-1B and ARK-2 cells. We also found that the combination of PFK158 and CBPt/Cis induced apoptosis- and autophagy-mediated cell death through inhibition of the Akt/mTOR signaling pathway. Mechanistically, we found that PFK158 downregulated the CBPt/Cis-induced upregulation of RAD51 expression and enhanced CBPt/Cis-induced DNA damage as demonstrated by an increase in γ-H2AX levels in HEC-1B and ARK-2 cells, potentially revealing a means to enhance PFK158-induced chemosensitivity. More importantly, PFK158 treatment, either as monotherapy or in combination with CBPt, led to a marked reduction in tumor growth in two chemoresistant EC mouse xenograft models. These data suggest that PFKFB3 inhibition alone or in combination with standard chemotherapy may be used as a novel therapeutic strategy for improved therapeutic efficacy and outcomes of advanced and recurrent EC patients.

## Introduction

Endometrial cancer (EC) is the most common gynecologic malignancy in developed countries [1], with an estimated 65,620 new cases and 12,590 deaths from EC in 2020 [2]. EC type I (endometrioid) are mostly low grade, estrogen positive with a good prognosis, and type II (predominantly papillary serous and clear cell) are high grade, usually occurs in older women and have a poor prognosis [3]. Although most EC is effectively treated with surgery, chemotherapy with platinum-based drug(s), the response rates for advanced or recurrent disease are low [1, 4, 5]. Therefore, there is a pressing need for more effective therapies aimed to overcome chemoresistance and improve the efficacy of EC treatments.

The upregulation of glycolysis is one of the major metabolic pathways implicated in cancer progression. One of the rate-limiting steps of glycolysis involves Fructose 2,6-bisphosphate (F-2,6-BP) and is mediated by 6-phosphofructo-2-kinase/fructose-2,6-biphosphatase 3 enzyme (PFKFB3). PFKFB3 catalyzes the synthesis of F2,6BP, which subsequently activates phosphofructokinase-1 (PFK-1) and upregulates the glycolytic flux [6]. Mounting evidence has shown that PFKFB3 expression is significantly higher in many cancers, including high-grade astrocytoma [7], head and neck squamous cell carcinoma [8], hepatocellular carcinoma [9], malignant pleural mesothelioma [10], breast and colon [11], gastric [12], thyroid [13], and ovarian cancer [14]. Furthermore, PFKFB3 plays an important role in regulating several cellular events, including pathological angiogenesis [15], carcinogenesis [6], cell cycle regulation [16], DNA repair[17], vessel sprouting [18], metastasis [19], and response to chemotherapy [14, 19]. Based on the regulatory function of PFKFB3 in glycolysis and cellular metabolism, an increasing number of studies have focused on investigating its role in tumor growth [8, 9]. Little is known about the role of PFKFB3 in EC and, thus, further studies are needed.

In this study, the antitumor effects of PFKFB3 inhibition in EC were evaluated in type I and type II chemoresistant EC cells in vitro and in vivo using two chemoresistant xenograft mouse models. We inhibited PFKFB3 by genetic silencing as well as chemically with the use of PFK158, a specific inhibitor of PFKFB3, and studied the impact of PFKFB3 inhibition on glycolysis, cell proliferation and chemoresistance in EC cells. Finally, the antitumor effects of PFK158 alone and in combination with chemotherapy on apoptosis, autophagy, DNA repair and the Akt/mTOR signaling pathway were examined.

## Results

### PFK158 treatment inhibits EC cell proliferation in vitro

**Fig. 1:**
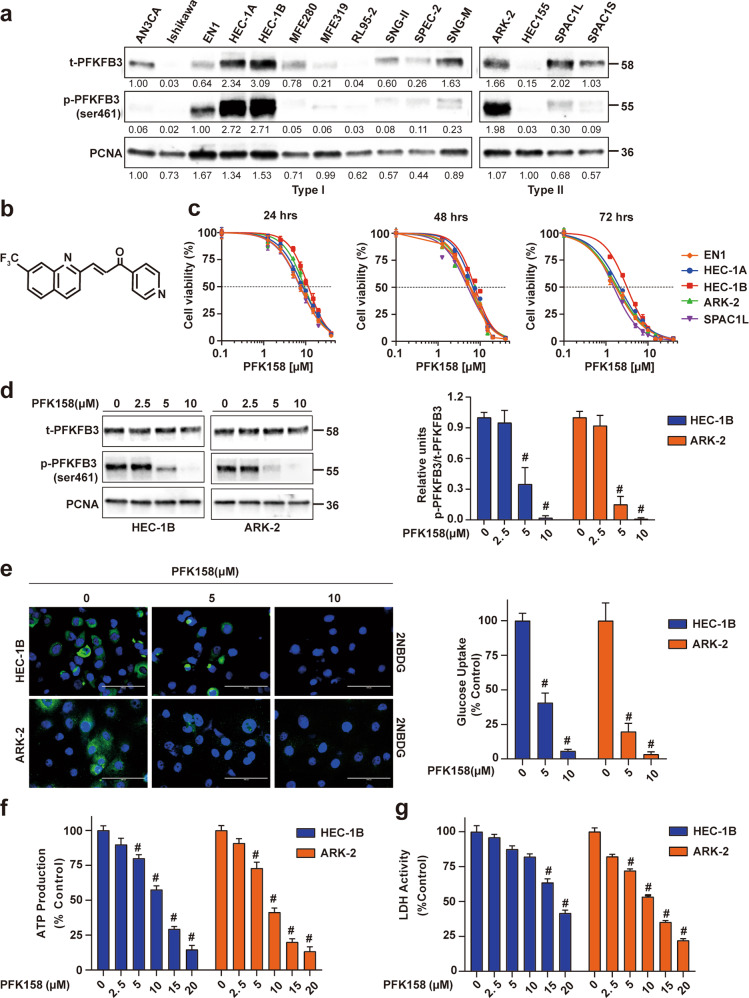
Pharmacological PFKFB3 inhibition shows obvious cytotoxicity and reduces aerobic glycolysis in vitro. **a** The expression levels of total (t)-PFKFB3 and phosphor (p)-PFKFB3 (ser461) in type I and type II EC cell lines were assessed by immunoblot analysis. **b** Chemical structure of PFK158, a PFKFB3 inhibitor. **c** EC cells (EN1, HEC-1A, HEC-1B, ARK-2, SPAC1L) were exposed to the indicated concentrations of PFK158 for 24, 48, or 72 h. Cell viability was analyzed by MTT assays and data are presented as mean ± SD. A minimum of three independent experiments were performed. **d** Expression of (t)-PFKFB3 and phosphor (p)-PFKFB3 (ser461) in HEC-1B and ARK-2 cells after PFK158 (0, 2.5, 5, 10 μM) treatment for 24 h were determined by immunoblotting (left panel). Band intensities were quantified and are presented as bar graphs (right panel). (*n* = 3; # vs control, ^#^*p* < 0.0001). **e** Fluorescence images of glucose uptake using 2-NBDG in HEC-1B and ARK-2 cells. Scale bar, 100 μm. Intracellular ATP generation (**f**) and LDH activity (**g**) were measured in HEC-1B and ARK-2 cells in the presence and absence of PFK158. All experiments were repeated at least three times. Data are shown as mean ± SD (*n* = 3; **p < 0.01, ^#^*p* < 0.0001).

We recently reported that activated PFKFB3 levels are high in ovarian cancer [14] and malignant pleural mesothelioma [10]. The expression levels of both total and phospho-PFKFB3 (PFKFB3^ser461^) were determined in both type I and type II EC cell lines. Among the EC cells tested, significant expression of p-PFKFB3 was observed in EN1, HEC-1A, HEC-1B (type I), ARK-2 and SPAC1L (type II) cell lines. Western blot analysis of chemoresistant HEC-1B and ARK-2 cells showed significantly higher levels of both t-PFKFB3 and p-PFKFB3 than the chemosensitive Ishikawa and RL95-2 cells (Figs. 1a and S1a, b). To investigate the ability of PFK158 (Fig. 1b), a selective inhibitor of PFKFB3, to inhibit EC cell proliferation in vitro, we exposed EC cell lines to a range of PFK158 concentrations (0–20 μM) for 24–72 h and assessed cell viability using MTT assays. PFK158 suppressed cell viability in a dose- and time-dependent manner in EC cells (Figs. 1c and S1c). PFK158 also showed a concentration-dependent decrease in p-PFKFB3 by immunoblot analysis in both HEC-1B and ARK-2 cell lines (Fig. 1d).

### Inhibition of PFKFB3 with PFK158 reduces glucose uptake, intracellular ATP and LDH activity in vitro

To evaluate whether PFK158 exhibits a reduction in glycolytic rate, we determined glucose uptake, intracellular ATP level, and lactate dehydrogenase activity in HEC-1B and ARK-2 cell lines in the presence and absence of different concentrations of PFK158. 2-NBDG was used to determine glucose uptake after PFK158 treatment for 24 h in HEC-1B and ARK-2. Results revealed that PFK158 reduced glucose uptake dose-dependent manner (Fig. 1e). Along with the decrease in glucose uptake, PFK158 showed a reduction in intracellular ATP levels (Fig.1f) and lactate dehydrogenase activity (Fig. 1g), suggesting PFK158 inhibits the glycolytic rate in EC cells. In addition, PFK158 downregulates Glut1 in EC cells (Fig. S2).

### Treatment with low doses of PFK158 and carboplatin/cisplatin has a synergistic effect in inhibiting cell viability in vitro

**Fig. 2:**
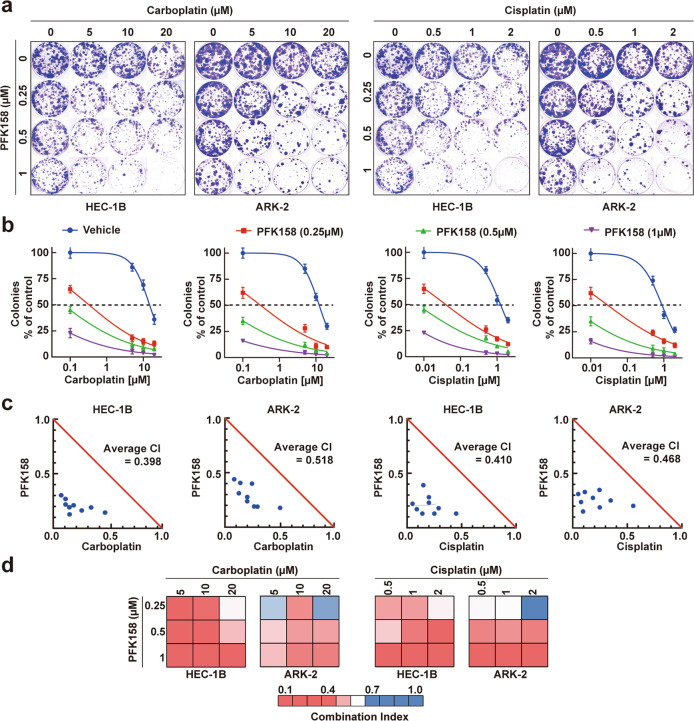
PFKFB3 inhibition shows synergistic activity with chemotherapeutic agents to inhibit cell proliferation in EC cells. **a**, **b** HEC-1B and ARK-2 cells were incubated with PFK158, CBPt/Cis at a range of concentrations, both individually and in combination for 72 h, and cell viability was measured by clonogenic assays. The synergistic inhibitory effect of PFK158 and CBPt/Cis combination is shown in HEC-1B and ARK-2 cells. All experiments were performed in triplicate. Representative images of plates are shown. **c**, **d** Normalized isobologram (obtained with Calcusyn software) over a range of PFK158 and CBPt or Cis dosage combinations. The combination index (CI) was plotted as a function of dose combination, with average CIs for the drug combination reported in the panel. The additive isobole is depicted in this panel as a red straight line, with synergistic dose combinations labeled below the isobole. An average CI of 1 indicates an additive effect, CI < 1 a synergistic effect, and CI > 1 an antagonistic effect.

While PFK158 and CBPt/Cis were cytotoxic when used independently, we next investigated the synergistic effect of combing PFK158 with CBPt/Cis by colony formation assays (CFA). Compared with the cells treated with the single agents, HEC-1B and ARK-2 cells treated with the combination regimen showed synergistic responses (Fig. 2a, b). Furthermore, the combination index (CI) values were used to verify the synergistic antitumor effects of PFK158 and CBPt/Cis. CompuSyn software was used to calculate CI values based on mean cell death, shown as Chou-Talalay plots for HEC-1B and ARK-2. Notably, the combination regimen showed strong synergy in HEC-1B and ARK-2 cells, with average CI values ranging from 0.398 to 0.518. (Fig. 2c, d)

### The combination of PFK158 and carboplatin/cisplatin promote apoptosis in EC cells

**Fig. 3:**
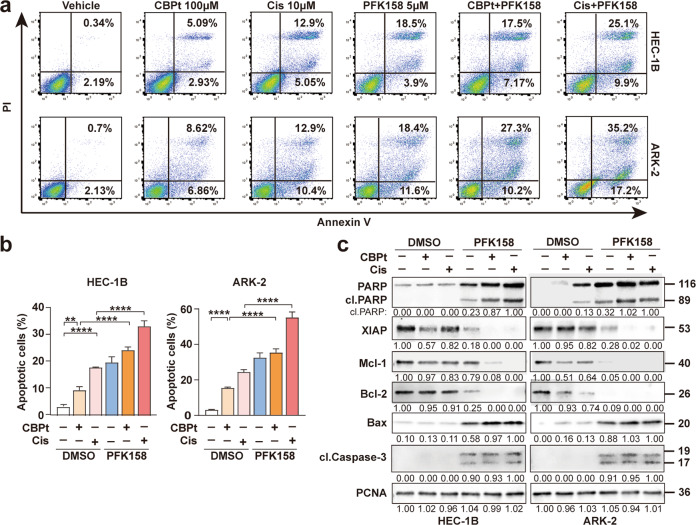
Cell apoptosis induced by PFK158 and carboplatin/cisplatin in EC cells. **a** HEC-1B and ARK-2 cells were treated with CBPt(100 μM)/Cis(10 μM), PFK158 (5 μM), or their combination for 24 h. The apoptotic cells were detected with Annexin-V/propidium iodide (PI) staining and analyzed by flow cytometry. Representative flow plots are shown. **b** Annexing-positive cells were defined as apoptotic. The data represent as mean ± SD (*n* = 3; **p < 0.01, ****p < 0.0001). **c** HEC-1B and ARK-2 cell were treated with PFK158 (5 μM), CBPt (100 μM)/Cis (10 μM) ± PFK158 for 24 h, and then the levels of PARP, cleaved caspase 3, XIAP, Mcl-1, Bcl-2 and Bax protein were determined by immunoblotting. PCNA served as a loading control.

To determine whether the synergistic growth inhibition induced by the combination of PFK158 and CBPt/Cis was due to apoptosis, apoptotic cells were scored by flow cytometry analysis using Annexin V and PI labeling. The results showed that co-treatment with PFK158 and CBPt /Cis led to a significant increase in the percentage of apoptotic cells in HEC-1B and ARK-2 (Fig. 3a, b). Furthermore, Western blot analysis revealed that the active form of PARP was significantly increased upon co-treatment, compared to single treatment alone, further demonstrating that the combination treatment enhances cell apoptosis. The levels of active Caspase 3 were increased after PFK158 and combination treatments in HEC-1B and ARK-2 cells. Meanwhile, we observed the upregulation of Bax and reduction of several anti-apoptotic Bcl-2 proteins, including Mcl-1 and X-linked inhibitor of apoptosis protein (XIAP). These findings suggest that co-treatment with PFK158 and CBPt/Cis promote apoptosis via the regulation of mitochondrion-related proteins (Fig. 3c).

### PFK158 alone and in combination with carboplatin/cisplatin significantly induce autophagic flux in EC cells

**Fig. 4:**
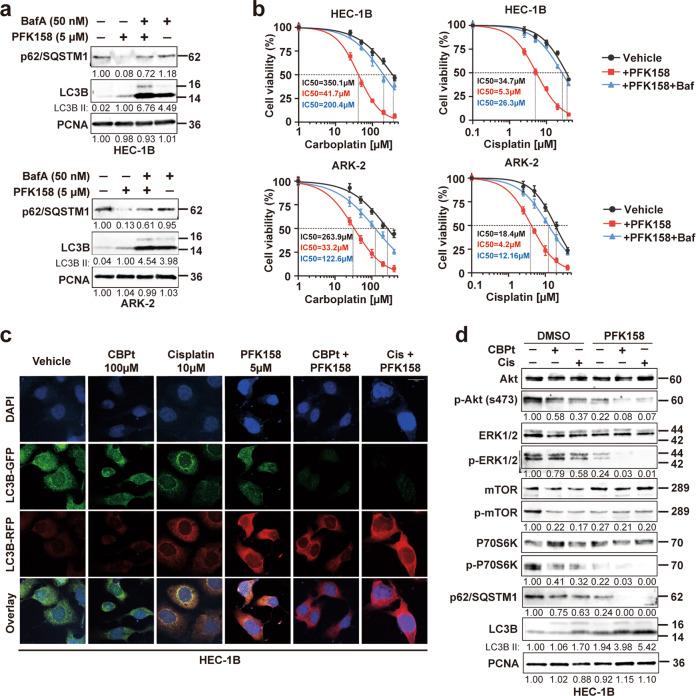
Combined effects of PFK158 and carboplatin/cisplatin on autophagy flux and the Akt/mTOR signaling pathway. **a** Western blot analysis of autophagy-related proteins (p62/SQSTM1 and LC3B) in HEC-1B and ARK-2 treated with PFK158 (5 μM) for 24 h in the presence or absence of bafilomycin A (50 nM) treatment for the last 12 h. PCNA was used as the loading control. **b** Cell viability assays were performed with a combination of increasing concentrations of CBPt/Cis with 5 μM PFK158 with and without bafilomycin A (BafA) pretreatment. Cells were pretreated with 50 nM BafA for 2 h followed by drug treatment. Cell viability was assessed by MTT assays 48 h later. The data are presented as mean ± SD. A minimum of three independent experiments were performed. **c** After transient expression of Cherry-GFP-LC3B (48 h), HEC-1B cells were treated with PFK158 (5 μM), CBPt (100 μM)/Cis (10 μM), or their combination for 24 h. Autophagic flux after treatment was investigated by confocal microscopy. Scale bar, 10 μm. **d** HEC-1B cells were treated with PFK158 (5 μM), CBPt (100 μM)/Cis (10 μM) ± for 24 h. Then, the cells were collected to assess the expression levels of autophagy-related proteins (Akt, p-Akt, mTOR, p-mTOR, p62 and LC3B). PCNA was used as the loading control.

Next, we sought to investigate whether PFK158 or in combination with CBPt/Cis could induce autophagy. Moreover, we measured the expression of the autophagic protein LC3BII, which is cleaved from the LC3BI protein during autophagosome formation and the levels of p62, which is degraded during autophagosome formation. The results showed autophagy was activated after PFK158 treatment in a dose-dependent manner, as confirmed by LC3BII upregulation and p62 downregulation in PFK158-treated HEC-1B and ARK-2 cells (Fig. S3a). Also, we observed that PFK158-induced autophagy was inhibited in the presence of Bafilomycin (BafA) in EC cells, as confirmed by the rescue of p62 and LC3BII levels (Fig. 4a). In addition, the cell viability assays showed that pretreatment with BafA resulted in increased resistance to CBPt/Cis in EC cells compared to cells not treated with BafA (Fig. 4b). These results clearly suggest that the PFK158-induced autophagy sensitizes EC cells to CBPt/Cis-induced cytotoxicity. Autophagic flux was also evaluated using a fluorescent-tagged Cherry-GFP-LC3B construct following autophagy induction [20]. Since the pH-sensitive GFP signal is quenched in lysosomes, the retention of the Red signal signifies increased drug-induced autophagic flux. In HEC-1B and ARK-2, the mCherry positive signals were significantly increased in PFK158 treated cells compared to vehicle and CBPt/Cis treated cells. Moreover, autophagic flux was also increased dramatically with the combination treatment suggesting that PFK158 potentiates the effect of CBPt/Cis in an autophagy-dependent manner (Figs. 4c and S3b).

### Co-treatment with PFK158 and carboplatin/cisplatin synergistically downregulate activation of the Akt/mTOR signaling in EC cells

Since the Akt/mTOR signaling pathway has been implicated in the pathogenesis of EC [21], we wondered whether PFK158 and CBPt/Cis combination would induce EC cell death through this signaling pathway. To determine the combined effects of PFK158 and CBPt/Cis on Akt/mTOR signaling proteins, HEC-1B and ARK-2 cells were treated with PFK158, CBPt/Cis, separately and in combination for 24 h. Western blot result showed that PFK158 decreased phosphorylation of Akt, mTOR and ERK1/2 in a dose-dependent manner (Fig. S3a). Treatment with CBPt/Cis alone just slightly reduced phosphorylation of Akt and ERK1/2. Co-treatment with PFK158 and CBPt/Cis led to a further reduction in phosphorylation of the Akt/mTOR and ERK1/2 as compared to each agent alone (Figs. 4d and S3c). The results demonstrate that a combination of PFK158 and CBPt/Cis inhibited transduction of the Akt/mTOR signaling pathways more effectively than treatment with either drug alone, indicating that these drugs acted synergistically to enhance suppression of the Akt/mTOR pathway.

### PFKFB3 knockdown reduces chemoresistance of endometrial cancer cells

**Fig. 5:**
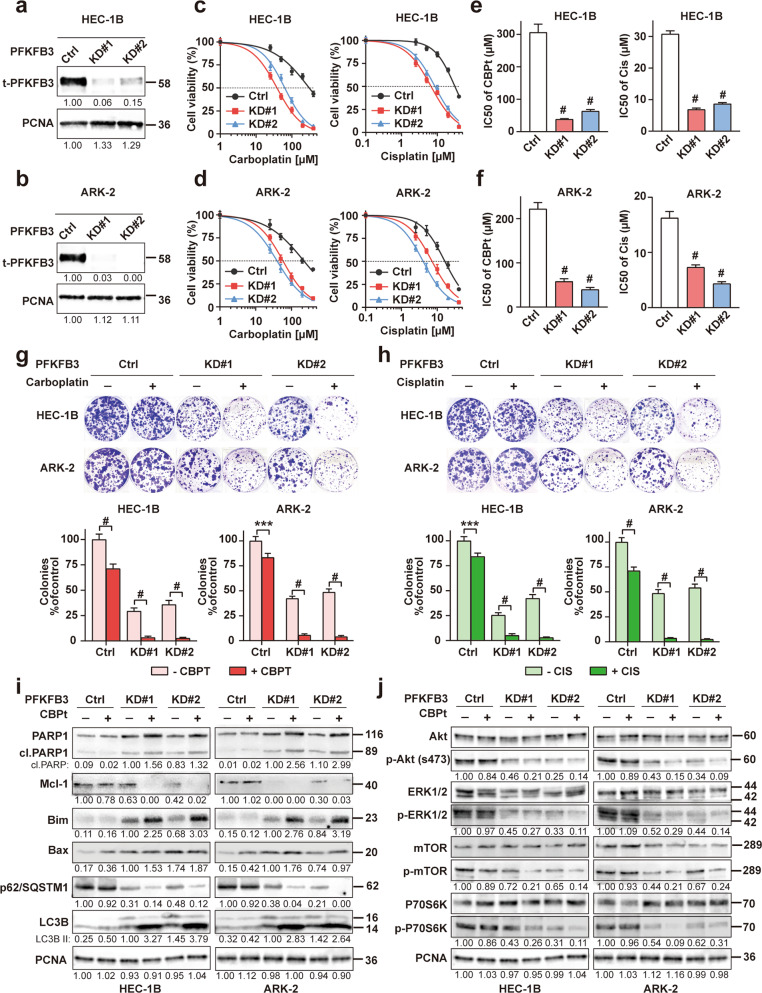
PFKFB3 knockdown enhances the chemosensitivity in EC. **a**, **b** PFKFB3 was knocked down by CRISPR/Cas9 in HEC-1B and ARK-2 cells. Western blot analysis of PFKFB3 expression was used to examine the effects of PFKFB3-KD in these cells. PCNA was used as a loading control. **c, d** MTT assays were performed to measure the sensitivity of these cells to chemotherapeutic drugs. Cells were exposed to various doses of carboplatin (CBPt) or cisplatin (Cis) for 48 h after plating. Chemosensitivity represented by IC50 values (**e**, **f**) for these cell lines were calculated using GraphPad Prism 7. The data represent as mean ± SD (*n* = 5, ^#^*p* < 0.0001). **g**, **h** HEC-1B and ARK-2 cells after PFKFB3 knockdown (KD) were treated with or without CBPt (5 μM)/Cis(0.5 μM) for 72 h. Colony formation assay was determined by crystal violet staining. The colony survival rate was calculated using the ImageJ software. The data represent as mean ± SD (*n* = 3, ***p < 0.001, ^#^*p* < 0.0001). **i**, **j** HEC-1B and ARK-2 cells were treated with or without CBPt (100 μM) for 24 h after PFKFB3 knockdown (KD) and the recognized biomarkers for cell apoptosis, autophagy and Akt/mTOR pathway were determined by Western blot analysis. PCNA was used as the loading control.

To further investigate whether PFKFB3 levels affected the sensitivity of EC cells to chemotherapy, we generated PFKFB3 knocked down (KD) clones in chemoresistant HEC-1B and ARK-2 cell lines using CRISPR/Cas9 targeting PFKFB3 with scrambled CRISPR/cas9 vector as controls and overexpressed (OE) PFKFB3 in chemosensitive cells (Ishikawa) with vector-transfected cells as controls. Efficient KD of PFKFB3 in HEC-1B and ARK-2 cells was verified by western blot analysis (Fig. 5a, b). MTT assays were performed to measure the sensitivity of PFKFB3-KD and PFKFB3-OE cells to chemotherapeutic drugs (CBPt and Cis). When the concentrations of CBPt or Cis required to inhibit cancer cell growth by 50% (IC50) were compared with and without PFKFB3 KD, silencing PFKFB3 in chemoresistant HEC-1B and ARK-2 cell lines significantly reduced the average CBPt and Cis IC50s (Fig. 5c–f). Inversely, the chemosensitivity of Ishikawa cells was dramatically decreased after PFKFB3 was OE (Figs. S4–5). Colony formation assays (CFA) also suggested that the proliferation of CBPt/Cis-treated HEC-1B and ARK-2 cells was lower in which PFKFB3 was inhibited compared with cells treated with CBPt/Cis alone (Fig. 5g, h). To confirm whether the expression level of PFKFB3 has any impact on the aerobic glycolysis of chemoresistant cells, the glycolytic rate in HEC-1B and ARK-2 cells after PFKFB3 KD was measured. Results showed that PFKFB3 KD reduced glucose uptake, intracellular ATP levels and lactate dehydrogenase activity (Fig. S6). In addition, western blot analysis illustrated that CBPt-treated HEC-1B and ARK-2 cells in which PFKFB3 had been KD were more likely to undergo apoptosis, autophagy compared with those treated with CBPt alone (Fig. 5i, j). While as shown in Fig. S7, PFKFB3 KD and OE did not show significant differences in cell cycle arrest compared to the control.

### PFK158 induces DNA damage, downregulates carboplatin/cisplatin-induced RAD51 to disrupt DNA repair, and potentially promotes chemosensitivity

**Fig. 6:**
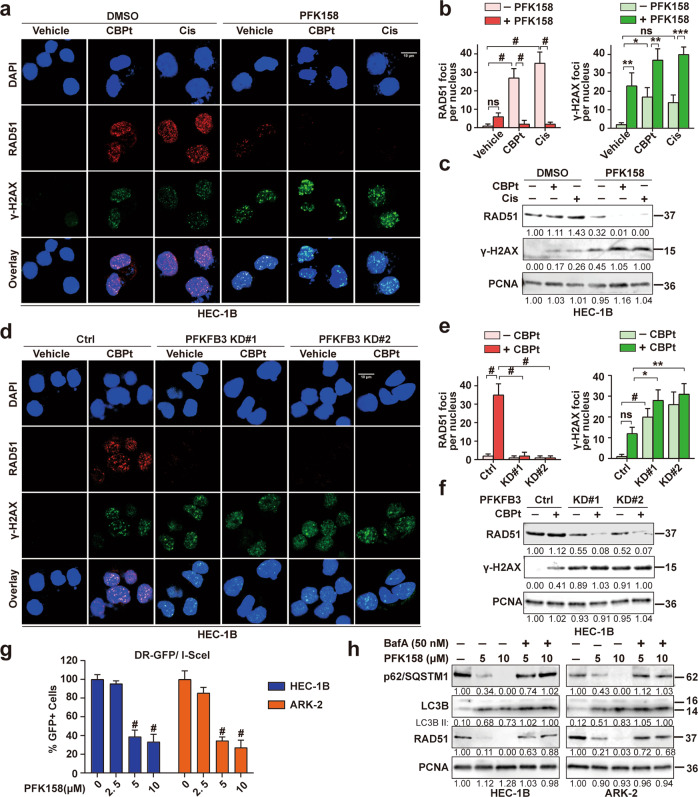
PFKFB3 inhibition induces DNA damage and downregulates CBPt/Cis-induced RAD51 to disrupt DNA repair in EC cells. **a** Confocal analysis of RAD51 foci (red) and γ-H2AX foci (green) in HEC-1B cells, following treatment with CBPt (100 µM)/Cis (10 µM), PFK158 (5 µM) or their combination for 24 h. *n* = 3 independent experiments. Scale bar, 10 μm. **b** Bar chart showing RAD51 foci (left panel) and γ-H2AX foci (right panel) as quantified using CellProfiler, *n* > 100 cells/treatment. The data represent as mean ± SD of three independent experiments, *p < 0.05; **p < 0.01; ***p < 0.001; ^#^*p* < 0.0001; ns not significant. **c** Western blotting was performed to determine the expression of RAD51 and γ-H2AX proteins in treated HEC-1B cells. PCNA was used as a loading control. These experiments were also done in HEC-1B cells after treatment with or without CBPt (100 µM) for 24 h after PFKFB3 KD (**d**–**f**). **g** I-SceI expression plasmid was transfected into HEC-1B and ARK-2 cells carrying DR-GFP, and the fraction of GFP positive cells after treatment with PFK158 (0, 2.5, 5, 10 μM) for 24 h were measured by flow cytometry, with untreated cells as control (100%). The data represent as mean ± SD (*n* = 3, ^#^*p* < 0.0001). **h** Western blot analysis of autophagy-related proteins (p62/SQSTM1 and LC3B) and RAD51 in HEC-1B and ARK-2 cells treated with PFK158 (5, 10 μM) for 24 h in the presence or absence of bafilomycin A (50 nM) treatment for the last 12 h. PCNA was used as the loading control.

Resistance to chemotherapy is multifactorial with previous studies that have focused on changes in the glutathione levels, increased activity of drug efflux transporters, altered drug targets playing a role in conferring chemoresistance [22]. In these events, enforced DNA damage response is one of the most important mechanisms for resistance to chemotherapy-based platinum agents [23]. Therefore, to better understand the mechanism underlying PFK158-induced chemosensitivity and synergy and based on the report that PFKFB3 plays a crucial role in promoting DNA repair [9, 17], we sought to determine if PFK158 treatment would modulate the expression levels of key DNA repair proteins. As shown in Figs. 6a–c and S8a–c, using both immunofluorescence and immunoblot analysis, we observed that while CBPt/Cis treatment-induced DNA damage as seen by an increase in γ-H2AX staining, PFK158 treatment enhanced CBPt/Cis-induced DNA damage in HEC-1B and ARK-2 cells. Whereas CBPt/Cis treatment induced higher levels of RAD51, a major repair protein involved in homologous recombination (HR) repair, in contrast, PFK158 promoted downregulation of RAD51. PFK158 decreased Rad51 and increased γ-H2AX in a dose-dependent manner in both HEC-1B and ARK-2 cell lines (Fig. S8d). The scenario was also recapitulated in PFKFB3-KD HEC-1B and ARK-2 cells with CBPt treatment compared to control cells (Figs. 6d–f and S8e–g). To better understand the consequence of PFK158 induced RAD51 downregulation, we checked if PFK158 treatment would inhibit HR repair compared to untreated cells. Using the well-established fluorescent reporter constructs (DR-GFP) in which a functional GFP gene will be reconstituted following HR event, we determined that PFK158 attenuated HR repair as demonstrated by a reduced percentage of GFP positive cells in the treated HEC-1B and ARK-2 cells compared to untreated control cells (Fig. 6g). In addition, we also tested if inhibiting autophagy would rescue RAD51 levels, similar to what we have shown with the rescue of p62 and LC3BII (Fig. 4a). Inhibiting autophagy with BafA rescued RAD51 levels in PFK158-treated cells implicating autophagy-mediated degradation of RAD51 (Fig. 6h) as a means to sensitize EC cells to chemotherapy.

### PFK158 alone and combined with carboplatin significantly inhibits tumorigenesis of subcutaneous xenograft tumors in vivo

**Fig. 7:**
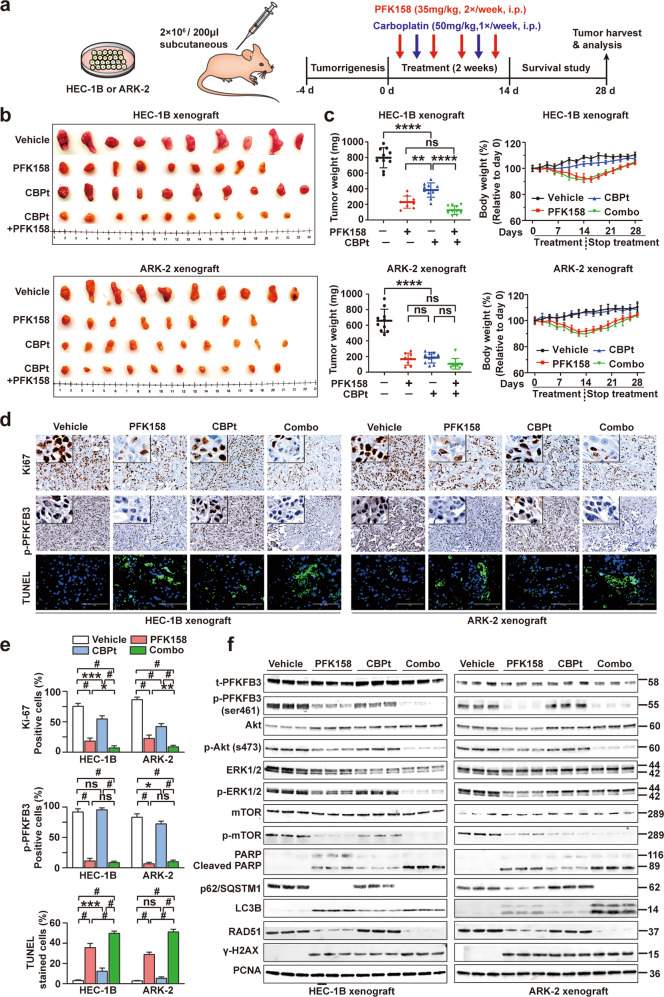
Antitumor efficacy of PFK158 alone and in combination with carboplatin in two mouse xenograft models. **a** Schematic diagram displaying the time course of tumor induction and treatment in mice. **b** Representative xenograft tumor images from each group are shown. **c** Effect of single-agent and dual treatment of PFK158 and CBPt on tumor growth of HEC-1B and ARK-2 cells in nude mice (*n* = 8–10 per group). Final tumor weights from different groups at the time of sacrifice are shown (left panel). The changes in the mouse body weights during the experiment were recorded (right panel). **d** IHC analysis for evaluating the expression of Ki67 and p-PFKFB3 in the tumors from vehicle or treated mice. Tumor tissues were also stained with TUNEL (green) and DAPI (blue) to investigate cell apoptosis. Scale bar, 100 μm. **e** Quantitative analyses of Ki67, p-PFKFB3, TUNEL staining in mouse xenograft tissues across treatment groups. Each bar represents the mean ± SD of three independent experiments, *p < 0.05, **p < 0.01, ***p < 0.001 and ^#^*p* < 0.0001 for the indicated comparison; ns not significant. **f** Western blot analysis was performed to assess t-PFKFB3, p-PFKFB3, Akt, p-Akt, ERK1/2, p-ERK1/2, mTOR, p-mTOR, PARP, p62, LC3B, RAD51 and γ-H2AX expression in lysates from the xenografts in each group with PCNA as a loading control. Western blot was carried out in triplicate per treatment.

To investigate whether our in vitro findings could be translated in an in vivo setting, two mouse xenograft models were developed using HEC-1B and ARK-2 cell lines. 2 × 10^6^ HEC-1B and ARK-2 cells were subcutaneously injected. Following the detection of palpable tumors, the mice were treated with vehicle, PFK158 alone, CBPt alone, or both for 14 days (Fig. 7a). A significant reduction of tumor growth, tumor volume and tumor weight was observed at day 28 in both PFK158 alone and combination groups. Vehicle groups exhibited a relatively constant rate of tumor growth throughout the observation period. In contrast, during the active treatment period and the post-treatment period, xenografts from treatment groups were relatively stable in size, especially PFK158 alone and combination groups. To assess how well PFK158 was tolerated, the body weight of mice was measured. Almost a 10% reduction in weight was observed in PFK158 alone and combination groups. But the body weight loss was reversible. (Figs. 7b, c and S9a, b).

H&E staining results demonstrated that PFK158- and combination treatment significantly increased tumor necrosis compared to control (Fig. S9c, d). PFK158 treatment alone or in combination with CBPt decreased p-PFKFB3 and Ki-67 expression compared to controls. Also, the detection of apoptosis by TUNEL staining showed a significantly higher number of TUNEL-positive cells in PFK158 only and combination groups (Fig. 7d, e), suggesting that PFK158 and combination may result in enhanced antitumor activity in vivo. Consistent with the in vitro data, western blot analysis in lysates from xenografts revealed decreased expression of p-PFKFB3, p-Akt, p-mTOR, p-ERK1/2, p62, RAD51 increased levels of cleaved PARP, LC3BII, γ-H2AX in PFK158 alone and combination treatment groups. (Fig. 7f).

## Discussion

**Fig. 8:**
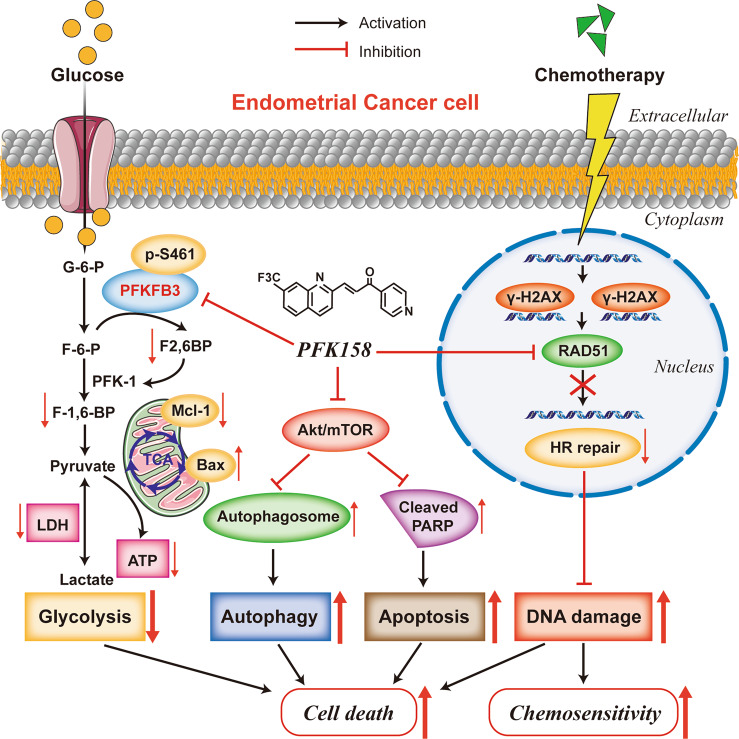
A working model that PFKFB3 inhibition plays a key role enhancing cell death and chemosensitivity in endometrial cancer. PFKFB3 inhibition led to the inactivation of the Akt/mTOR signaling pathway, induced apoptosis and autophagy, disrupted DNA Repair which consequently enhanced cell death and chemosensitivity.

In this study, we demonstrated that PFKFB3 overexpression is associated with chemoresistance in EC. The combination of PFK158 with CBPt or with Cis acted synergistically to significantly decrease cell viability in vitro and inhibit tumor growth in two chemoresistant EC mouse xenograft models in vivo through induction of apoptosis, autophagy, disrupting DNA repair, and inactivation of the Akt/mTOR signaling pathway in EC (Fig. 8).

Metabolic reprogramming has been widely accepted as a distinctive hallmark of cancer. Cancer cells exhibit increased glucose uptake, enhanced glycolysis and lactate production, and reduced oxygen consumption, even in oxygen-rich conditions, known as the Warburg effect or aerobic glycolysis [24–26]. In addition to promoting tumorigenesis, aerobic glycolysis plays a vital role in developing drug resistance [27]. Drugs designed to target cancer metabolism are being developed to halt cancer survival, progression and metastasis [28]. PFKFB3 has been recognized as an important metabolic target in cancer. Recent findings indicate that inhibition of PFKFB3, either with chemical inhibitors or genetic silencing, reduces the glycolytic rate and suppresses tumor cell proliferation [29]. In a previous study from our group, we found that inhibition of PFKFB3 with PFK158 promotes lipophagy and autophagy and, importantly, enhances the efficacy of conventional chemotherapy in ovarian cancer [14].

Studies have shown that intricate crosstalk between apoptosis and autophagy is critical in determining cell fate [30, 31]. These reports attest to the well-established notion that autophagy can either play a pro-survival or a pro-death role, depending on cell type and treatment characteristics. Most studies have shown that autophagy promotes chemoresistance, and inhibiting autophagy may increase cancer cell chemosensitivity [32]. However, some studies also reported that autophagy induction sensitizes cells to chemotherapy [33–35]. Although a few recent studies have provided some data on the role of autophagy following PFKFB3 inhibition treatment in cancer, the relationship between PFKFB3 and autophagy in cancers remains controversial. Yan et al. found that inhibition of PFKFB3 suppresses autophagy and enhances cytotoxicity in colorectal cancer cells [36]. However, in ovarian cancer cells, PFKFB3 inhibition has been reported to lead to autophagy induction and chemosensitivity [14]. Consistent with the results in ovarian cancer, we found in our study that both autophagy and apoptosis were increased following PFKFB3 inhibition in EC. Also, inhibition of autophagy by BafA significantly decreased the combined PFK158 plus CBPt/Cis-induced cytotoxicity in EC cells. Thus, targeting PFKFB3 in EC with PFK158 enhanced the apoptosis- and autophagy-mediated cell death caused by chemotherapy.

The impact of PFKFB3 inhibition on the Akt/mTOR pathway has not been previously described. In this study, we discovered for the first time that PFK158 in combination with either CBPt or Cis synergistically downregulated activation of the Akt/mTOR signaling pathway both in vitro and in vivo. The Akt/mTOR signaling pathway is frequently overactive in women with EC. It has been associated with aggressive disease and poor prognosis, accounting for cell proliferation, apoptosis, autophagy, and chemoresistance of EC cells [21, 37, 38]. It has been vigorously pursued as a target for drug development leading to a number of ongoing clinical trials with various mTOR inhibitors currently in this patient population. Specifically, the Akt inhibitor MK2206 [39] and mTOR inhibitors Ridaforolimus [40], Everolimus [41], and Temsirolimus [42] are under investigation in phase 2 trials for patients with EC. The challenge in targeting the PI3K/Akt/mTOR pathway stems from the presence of complex feedback loops within the signaling cascade leading to activation of compensatory pathways or shift in isoform dependency [43, 44]. Regimens combining drugs that target different parts of the PI3K/Akt/mTOR pathway may result in a greater therapeutic benefit compared to when using each drug alone. Thus, combining PFK158 with one of the existing Akt/mTOR inhibitors could be a novel therapeutic approach for EC patients who did not derive clinical benefit from monotherapy with Akt/mTOR pathway inhibitor.

In cancer cells, increased DNA repair capacity can reverse the DNA damage, which is caused by chemotherapeutic drugs such as CBPt and Cis, and confer resistance against these drugs [23]. In this context, previous studies have highlighted that increased expression of the major DNA repair protein RAD51 is associated with resistance to chemotherapy in several different tumor types [45–47]. The inhibition of RAD51 will disrupt DNA repair by HR and improve response to chemotherapy treatments [48]. In this study, we show that pharmacological and genetic inhibition of PFKFB3 suppressed CBPt/Cis-mediated upregulation of RAD51, suggesting that PFKFB3 inhibition probably reverses CBPt/Cis-induced DNA repair and regulates the γ-H2AX-RAD51 axis to overcome resistance to chemotherapy. In addition, our data that shows inhibiting autophagy with BafA rescues RAD51 levels both in EC cells lends additional support that autophagy-mediated cell death may have a role in PFK158-induced chemosensitivity through the modulation of DNA repair protein, RAD51 levels. In the future, in-depth studies in EC cells after RAD51 knockdown or overexpression are needed to further consolidate these preliminary studies.

Overall, our study is the first to investigate the impact of PFKFB3 inhibition on EC and the underlying mechanistic pathway associated with the related downstream effects, indicating a potential therapeutic benefit in targeting PFKFB3 with the use of PFK158 alone as well in combination with platinum-based treatment to enhance its efficacy, reverse chemoresistance and improve outcomes of patients with advanced or recurrent EC.

## Materials and methods

### Chemicals and reagents

PFK158 was obtained on an MTA from Gossamer Bio Inc (San Diego, CA). For in vitro experiments, PFK158 was dissolved in DMSO, and for in vivo experiments, PFK158 was dissolved in 40% solution of Captisol in ddH_2_O. Other reagents, antibodies are shown in Table S1.

### Cell lines and cell culture

Cell lines used in this article are presented in Supplementary Table S2. All cell lines were maintained in a humidified incubator at 37 °C with 5% CO_2_.

### CRISPRcas9-mediated knockdown and overexpression of PFKFB3

To construct PFKFB3 knockdown (KD) and overexpression (OE) in EC cell lines, HEC-1B, ARK-2 and Ishikawa cells were seeded at a density of 1 × 10^5^ cells/well into 6-well plates, and after 24 h, transfected with 5 μg PFKFB3 CRISPR KD/OE plasmid (All-in-one sgRNA clone for Human PFKFB3 gene, Vector: pCRISPR-CG01, Target site: CGGCTCTGCGTCAGTTCCAA, Genecopoeia, MD, USA) using 4.5 μl Attractene and up to 250 μl OptiMEM/well. Two days later, transfection efficiency was confirmed by fluorescence microscopy. Afterward, the transfected cells were selected with G418 for ~2 weeks before individual colonies were isolated. PFKFB3 knockdown and overexpression clones were verified by western blot for cell lysates.

### Western blot analysis

Western blot analysis was performed as previously described [10]. The blots were probed with indicated antibodies. The membrane was washed and scanned under the OdysseyFc Imaging system (Nebraska, USA). Quantitative analysis of protein expression was calculated using ImageJ software.

### Clonogenic assays

500 cells were plated onto 6-well plates and allowed to grow overnight. The medium was then supplemented with vehicle alone or the indicated drugs for 72 h. Then the medium was discarded. Following washing, fresh medium was re-added to the plates, and cells were incubated for up to 14 days until colonies became visible. Cells were washed twice with PBS and fixed with 100% methanol, stained with crystal violet, and washed with deionized water. The colonies were imaged and counted using ImageJ software.

### Cell viability assays

Cells were seeded into 96-well plates at a density of 3 × 10^3^ cells per well and incubated for 24 h. Cells were then treated with different doses of the compound for indicated times. The inhibitory concentrations 50% (IC_50_) values were determined by MTT assays, as previously described [10].

### Cell imaging using 2-NBDG

Glucose uptake of the live EC cells was measured using 2-NBDG, as previously described. Cells were treated with PFK158 (0, 5, 10 µM) for 24 h followed by the incubation with 2-NBDG (150 µg/ml) [Cayman Chemicals, Michigan, USA] for 30 min in the glucose-free medium. Subsequently, cells were washed, mounted, and analyzed in the Zeiss LSM510 fluorescence microscope. The fluorescent intensities were calculated using ImageJ software.

### ATP, LDH activity assay

1 × 10^6^ cells were seeded in 96-well plates overnight and treated with PFK158 (0–20 µM) for 24 h. According to the manufacturer’s protocol, the LDH released into the medium was transferred to a new 96-well plate and mixed with a reaction mixture (Pierce, USA). Measurement of mitochondrial ATP production experiment was performed according to the manufacturer’s (BioVision Inc. USA) protocol. LDH activity and ATP production were represented as a percentage of control by normalizing the OD values of untreated control cells.

### Synergy assays

To determine synergy, a range of drug concentrations was used, and the CI values were calculated using CompuSyn software using a non-constant ratio approach, according to Chou-Talalay [49]. The CI values were calculated; CI < 1 indicates a synergistic effect; CI < 0.7 indicates a significant synergistic effect. The values represent the mean ± SD of three independent experiments. CI > 1 represents an antagonistic effect.

### Apoptosis assay

Cells were plated in 6-well plates at a density of 1 × 10^6^ cells per well and allowed attaching overnight. Then cells were exposed to CBPt (100 μM), Cis (10 μM), PFK158 (5 μM), PFK158 + CBPt, PFK158 + Cis for 24 h. Afterward, floating and adherent cells were stained with Annexin V-Pacific blue and propidium iodide (5 μg/ml). Cells were analyzed by CellQuest Pro software (BD FACSCalibur) as previously described [50].

### Immunofluorescence (IFC) assay

HEC-1B/ARK-2 cells, untreated and treated with PFK158, transiently transfected with Cherry-GFP-LC3B for 48 h or PFKFB3 downregulated cells, were grown on four-well chambered slide for the desired time. After drug treatment, cells were washed with PBS, fixed with 4% formaldehyde, permeabilized using 0.2% Triton X-100 in PBS for 15 min. After blocking with 2% bovine serum albumin (BSA) in PBS for 1 h at room temperature, the cells were incubated overnight with primary antibodies (RAD51 and γ-H2AX) at 4 °C. The cells were then incubated with fluorescent secondary antibodies for 1 h at room temperature. IFC was performed as previously described [51]. And cells were visualized by using Zeiss-LSM 510 confocal microscope. Quantification of the fluorescence was measured using ImageJ software.

### DR-GFP (HR) assays

HEC-1B and ARK-2 cells were transfected with pDR-GFP, an HR substrate that generates a functional green fluorescent protein (GFP) upon successful HR by I-SceI cleavage [52]. 48 h later, the cells were transfected with pCβASceI plasmid and 24 h later treated with increasing concentrations of PFK158 for 24 h and then analyzed for GFP fluorescence by flow cytometry.

### Therapeutic study of the mouse xenograft tumor models

Animal experiments complied with the Institutional Animal Care and Use Committee (IACUC) guidelines at the Mayo Foundation, following approved protocols. Mice were injected with two ×10^6^ HEC-1B or ARK-2 cells subcutaneously into the right dorsal flanks. Treatment was initiated after 5-days post-inoculation when the subcutaneous implants measured 3 mm by 3 mm. Mice implanted with each cell line were randomly assigned to four treatment groups and treated for 2 weeks (*n* = 10 per group). Groups were treated as follows: (i) HEC-1B v) ARK-2 intraperitoneal injections of 40% Captisol for the control group; (ii) HEC-1B vi) ARK-2 intraperitoneal injections of PFK158 at 35 mg/kg twice weekly (35 mg/kg, 2×/week, i.p.) (iii) HEC-1B vii) ARK-2 intraperitoneal injections of CBPt at 50 mg/kg weekly (50 mg/kg, 1×/week, i.p.) (iv) HEC-1B viii) ARK-2 combination of CBPt (50 mg/kg, 1×/week, i.p.) and PFK158 (35 mg/kg, 2×/week, i.p.). Body weight and tumor size were measured every 2 days with a caliper, and tumor volume was calculated using the following formula: V = length × width2×(π/6) (length = the longest diameter, width = corresponding perpendicular diameter). At the end of the experiments, the mice were euthanized. Harvested tumors were weighed and processed for further analyses (fresh-frozen, formalin-fixed paraffin-embedded).

### Histological examination and immunohistochemistry

Tumor tissues were fixed in 10% neutral buffered formalin and embedded in paraffin. Then the samples were sectioned at 5 µm thickness and stained with hematoxylin and eosin (H&E). The expression levels of Ki-67 and p-PFKFB3 were evaluated by immunohistochemistry in the paraffin-embedded tumor sections, according to a previously described protocol [53].

### TdT-mediated dUTP-biotin nick end labeling (TUNEL) assay

Cell apoptosis in tumor tissues was performed by terminal dUTP Nick End-Labeling (TUNEL) staining with an In Situ Apoptosis Detection Kit according to the manufacturer’s instructions.

### Statistical analysis

Data are represented as mean ± SD deviation from at least three independent experiments. All statistical analyses were performed using the GraphPad Prism 7 software. Data were analyzed using either non-linear regression or *t*-test or one-way ANOVA as appropriate. The minimal level of significance was *p* = 0.05. *p < 0.05; **p < 0.01; ***p < 0.001; **** or ^#^*p* < 0.0001. If not specified, the analysis is not significant.

## Supplementary information

Supplemental Material
