# Supplementary material for: Inhibition of PFKFB3 induces cell death and synergistically enhances chemosensitivity in endometrial cancer
Source: Oncogene. 2021 Jan 8;40(8):1409–24. doi: 10.1038/s41388-020-01621-4 (PMC7906909; doi:10.1038/s41388-020-01621-4)
Supplement: Supplementary file 1 — Supplemental Material [file 41388_2020_1621_MOESM1_ESM.pdf]

Table S1 List of Antibodies and Reagents

Primary Antibodies	Catalog No.	Company
Akt	#4691	Cell Signaling Technology
Bax	#32503	Abcam
Bcl-2	#692	Abcam
Bim	#2819	Cell Signaling Technology
Cleaved Caspase 3	#9661	Cell Signaling Technology
Glut1	#7903	Santa Cruz Biotechnology
γ-H2AX	#26350	Abcam
LC3B	#3868	Cell Signaling Technology
Mcl-1	#4572	Cell Signaling Technology
mTOR	#2983	Cell Signaling Technology
p44/42 MAPK (Erk1/2)	#9102	Cell Signaling Technology
p62/SQSTM1	#48402	Santa Cruz Biotechnology
p70S6 Kinase	#2708	Cell Signaling Technology
p-p70S6 Kinase	#9204	Cell Signaling Technology
p-Akt (Ser473)	#4060	Cell Signaling Technology
p-mTOR	#5536	Cell Signaling Technology
p-p44/42 MAPK	#4370	Cell Signaling Technology
PARP	#9542	Cell Signaling Technology
PCNA	#9857	Santa Cruz Biotechnology
PFKFB3	#181861	Abcam
p-PFKFB3	Custom made	Genescript Inc
RAD51	#133534	Abcam
XIAP	#2042	Cell Signaling Technology
**Reagents**	**Catalog No.**	**Company**
2-NBDG	#186689-07-6	Cayman Chemicals
3-(4,5-dimethylthiazol-2-yl)-2,5-diphenyltetrazolium bromide (MTT)	M6494	ThermoFisher Scientific
ATP Colorimetric/Fluorometric Assay Kit	#K354	Biovision
Carboplatin	#55770169	TEVA UK limited
Cisplatin	#232120	Calbiochem
fetal bovine serum (FBS)	#S181A	Biowest
Lactate Dehydrogenase Activity Colorimetric Assay Kit	#K726	Biovision
Pacific BlueTM Annexin V	#640918	BioLegend
Propidium iodide	P1304MP	Thermo Fischer

Table S2 List of Cell Lines

Cell line	Base Media	Supplements
AN3CA	DMEM	10% FBS and 1% Pen/Strep
ARK-2	DMEM-F12	10% FBS and 1% Pen/Strep
EN1	DMEM	10% FBS and 1% Pen/Strep
HEC-1A	McCoy's 5a	10% FBS and 1% Pen/Strep
HEC-1B	DMEM-F12	10% FBS and 1% Pen/Strep
HEC155	DMEM	10% FBS and 1% Pen/Strep
Ishikawa	DMEM	10% FBS and 1% Pen/Strep
MFE280	DMEM	10% FBS and 1% Pen/Strep
MFE319	DMEM	10% FBS and 1% Pen/Strep
RL95-2	DMEM-F12	10% FBS and 1% Pen/Strep
SNG-II	DMEM	10% FBS and 1% Pen/Strep
SNG-M	DMEM	10% FBS and 1% Pen/Strep
SPAC-1L	RPMI-1640	10% FBS and 1% Pen/Strep
SPAC-1S	RPMI-1640	10% FBS and 1% Pen/Strep
SPEC-2	DMEM	10% FBS and 1% Pen/Strep

a

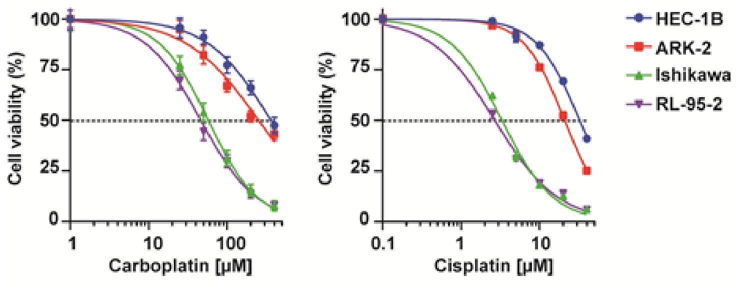

b

IC50s (μM)	HEC-1B	ARK-2	Ishikawa	RL-95-2
Carboplatin	360.80 ± 32.13	251.40 ± 24.79	57.48 ± 12.98	46.15 ± 13.20
Cisplatin	32.42 ± 4.32	20.87 ± 3.21	3.32 ± 1.21	2.68 ± 0.76

c

IC50s (μM)	EN1	HEC-1A	HEC-1B	ARK-2	SPAC1L
PFK158 24h	7.41 ± 1.54	8.53 ± 1.68	11.51 ± 2.30	9.32 ± 2.53	7.28 ± 1.49
PFK158 48h	5.70 ± 1.08	6.72 ± 1.75	7.50 ± 2.12	5.81 ± 1.02	5.11 ± 0.76
PFK158 72h	1.71 ± 0.54	2.12 ± 0.32	3.15 ± 0.21	1.86 ± 0.37	1.54 ± 0.18

Fig. S1 Effect of carboplatin and cisplatin in EC cell viability *in vitro*.

a EC cells (HEC-1B, ARK-2, Ishikawa, RL95-2) were exposed to increasing concentrations of carboplatin or cisplatin for 48h. Cell viability was analyzed by MTT assays and data are presented as mean ± SD. A minimum of three independent experiments were performed. IC50 values of carboplatin (CBPt)/cisplatin (Cis) (**b**) PFK158 (**c**) from EC cell lines were calculated using GraphPad Prism 7. The data represent as mean ± SD (n=5).

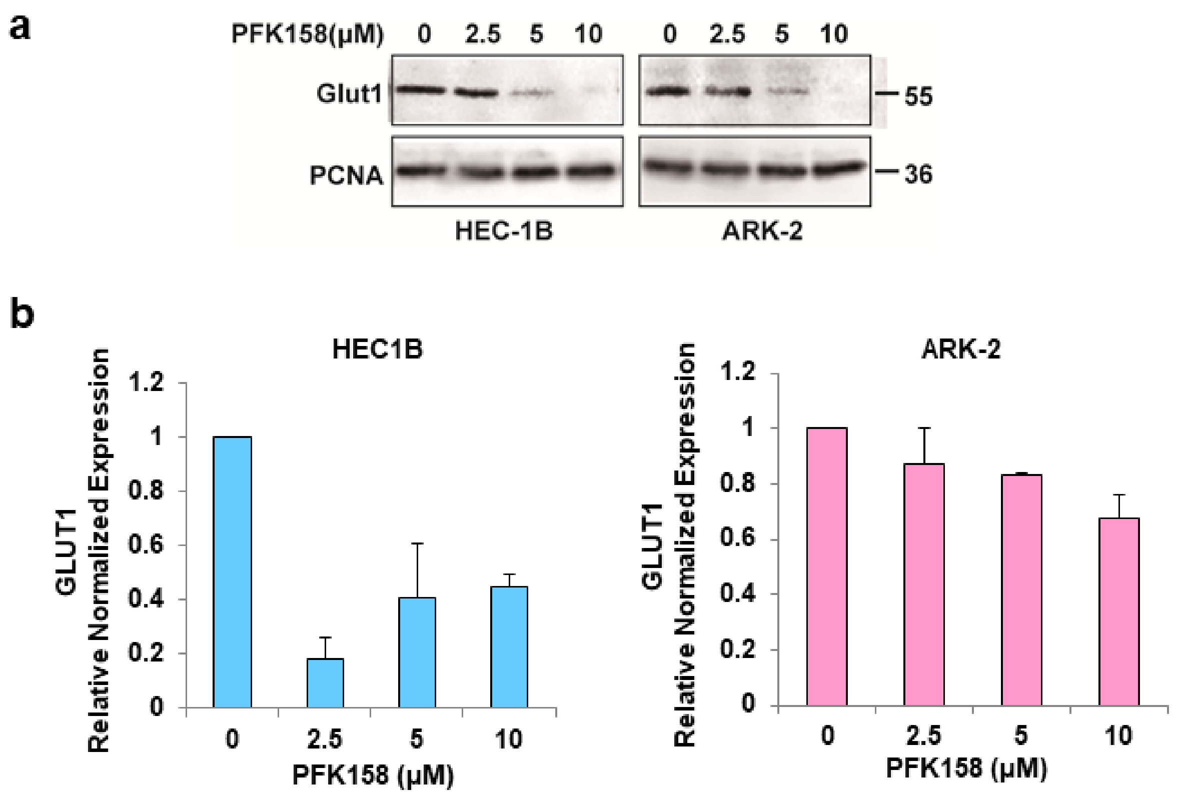

Fig. S2 PFK158 downregulates Glut1 in EC cells

HEC-1B and ARK-2 cells treated with PFK158 (0, 2.5, 5, 10μM) for 24h. Western blotting (**a**) and RT-PCR (**b**) were performed to determine the expression of Glut1 in treated cells. PCNA was used as a loading control.

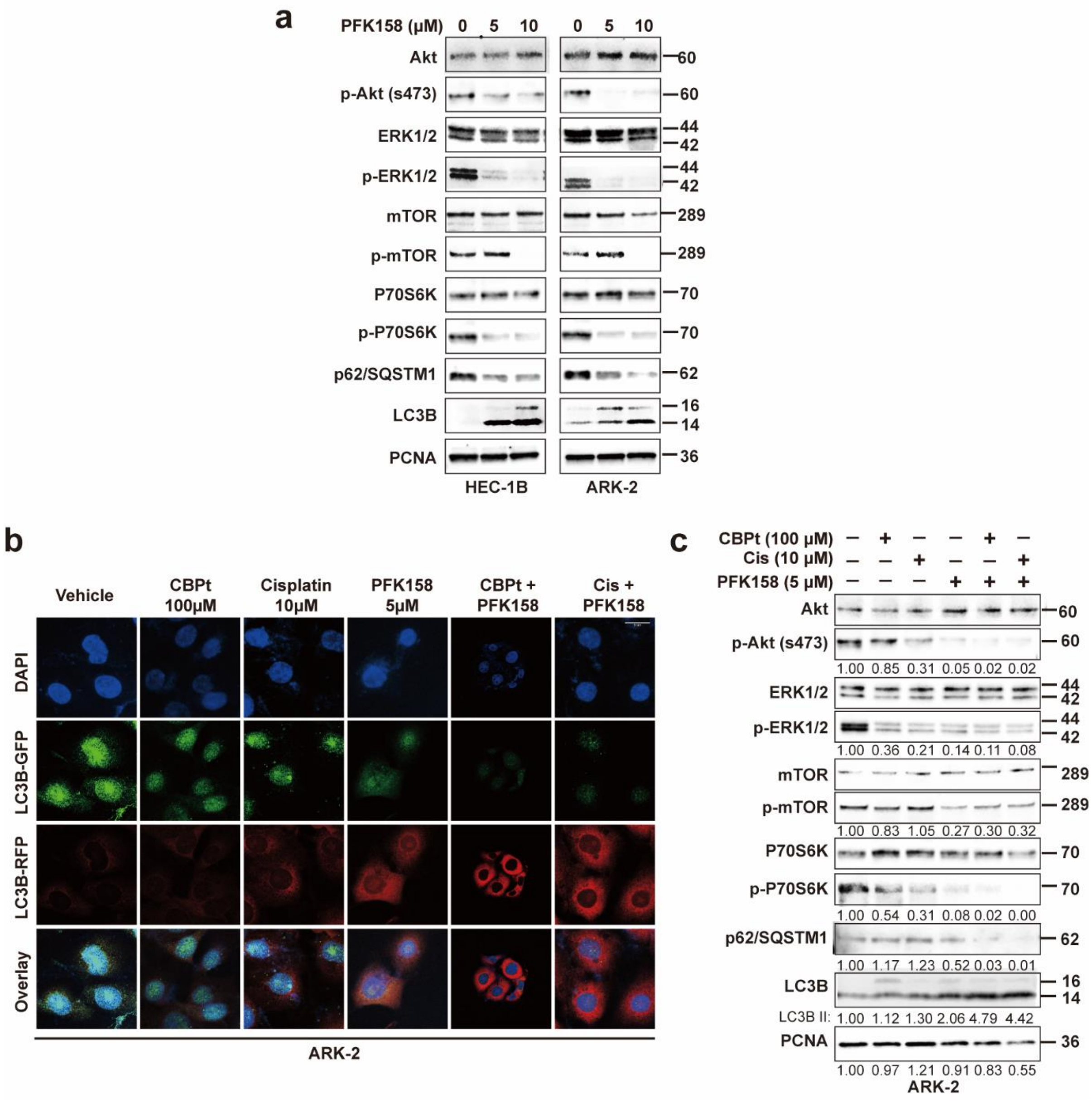

Fig. S3 (Related to Fig 4) Combined effects of PFK158 and carboplatin/cisplatin on autophagy flux and the Akt/mTOR signaling pathway

a PFK158 induces autophagy flux and inhibits the Akt/mTOR pathway in a dose-dependent manner in HEC-1B and ARK-2 cells. **b** After transient expression of Cherry-GFP-LC3B (48h), ARK-2 cells were treated with PFK158 (5μM), CBPt (100μM)/Cis (10μM), or their combination

for 24h. Autophagic flux after treatment was investigated by confocal microscopy. Scale bar, 10μm. **c** ARK-2 cells were treated with CBPt (100μM)/Cis (10μM) ± PFK158 (5μM) for 24h. Then, the cells were collected to assess the expression levels of autophagy-related proteins (Akt, p-Akt, mTOR, p-mTOR, p62 and LC3B). PCNA was used as the loading control.

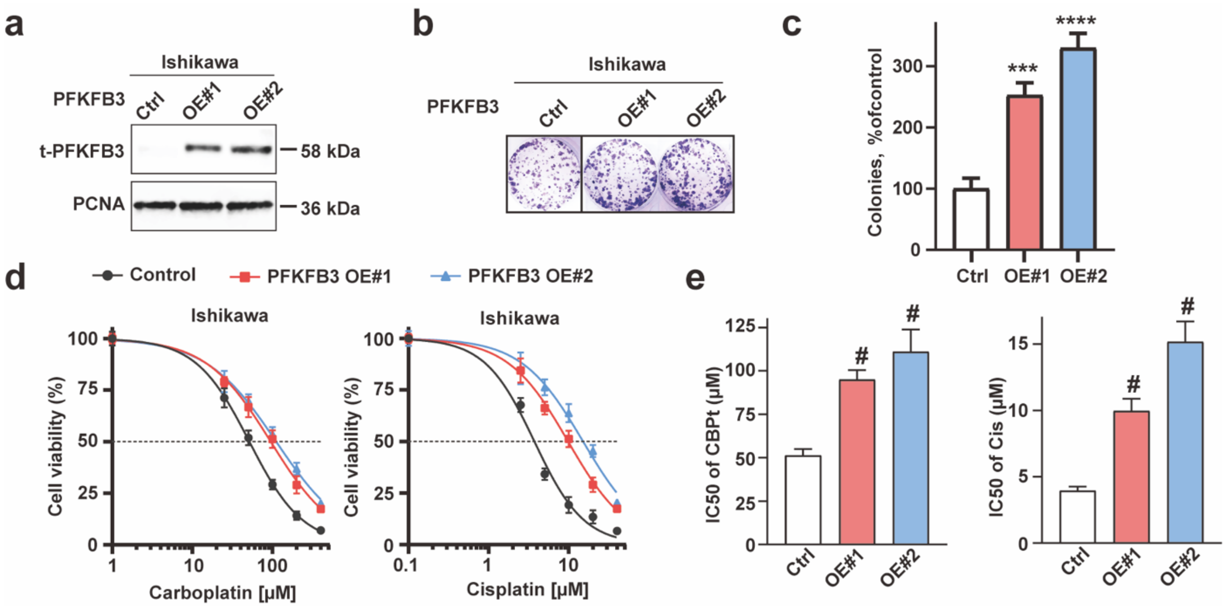

Fig. S4 (Related to Fig 5) Effects of PFKFB3 overexpression (OE) on cell viability and the sensitivity to carboplatin/cisplatin in Ishikawa cells.

a PFKFB3 was overexpressed (OE) by CRISPR/Cas9 in Ishikawa cells. Western blot analysis of PFKFB3 expression was used to examine the effects of PFKFB3-OE in the cells. PCNA was used as a loading control. **b, c** Cell viability was measured using clonogenic assays after

PFKFB3 OE in Ishikawa cell. Three independent experiments were performed. **d** MTT assays were performed to measure the sensitivity of Ishikawa cells to chemotherapeutic drugs after PFKFB3 OE. Cells were exposed to various doses of carboplatin (CBPt) or cisplatin (Cis) for 48h after plating. **e** Chemosensitivity represented by IC50 values for these cell lines were calculated using GraphPad Prism 7. The data represent as mean ± SD (n=5, #*p*<0.0001).

IC50s (μM)	HEC-1B		
	Ctrl	KD#1	KD#2
Carboplatin	305.98 ± 25.15	38.35 ± 1.93	63.22 ± 5.18
Cisplatin	30.80 ± 1.01	6.88 ± 0.43	8.68 ± 0.34

IC50s (μM)	ARK-2		
	Ctrl	KD#1	KD#2
Carboplatin	222.12 ± 14.34	58.00 ± 6.11	39.64 ± 4.57
Cisplatin	16.25 ± 1.18	7.32 ± 0.43	4.35 ± 0.35

IC50s (μM)	Ishikawa		
	Ctrl	OE#1	OE#2
Carboplatin	51.28 ± 3.68	94.91 ± 5.59	111.00 ± 12.84
Cisplatin	3.95 ± 0.30	9.95 ± 0.92	15.15 ± 1.55

Fig. S5 (Related to Fig 5) Summary of CBPt/Cis IC50 data from HEC-1B and ARK-2 cell lines upon PFKFB3 knockdown (KD), and Ishikawa cell line upon PFKFB3 overexpression (OE).

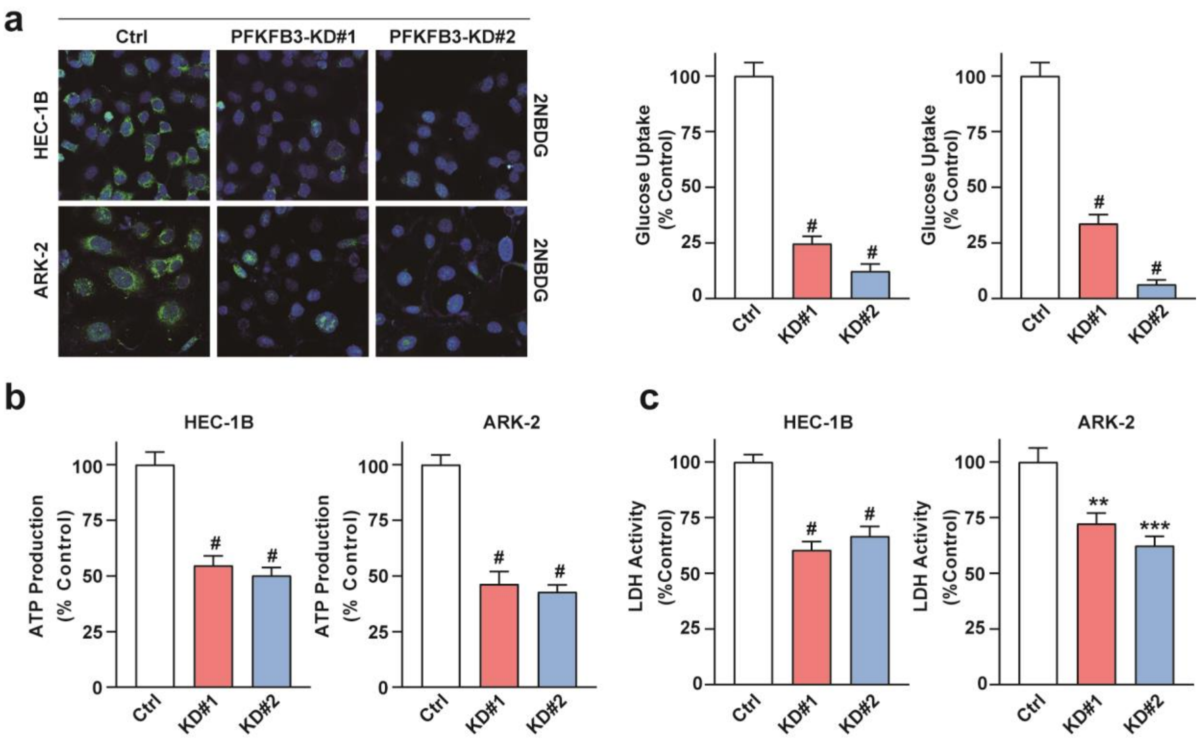

Fig. S6 Effects of PFKFB3 knockdown on aerobic glycolysis in EC cells.

a Fluorescence images of glucose uptake using 2-NBDG in HEC-1B and ARK-2 cells after PFKFB3 knockdown (KD) by CRISPR/Cas9. Intracellular ATP generation (**b**) and LDH activity (**c**) were measured in HEC-1B and ARK-2 PFKFB3-KD cells. All experiments were repeated at least three times. Data are shown as mean ± SD of three replicates per cell line. (n=3; ***p*<0.01; ****p*<0.001; #*p*<0.0001).

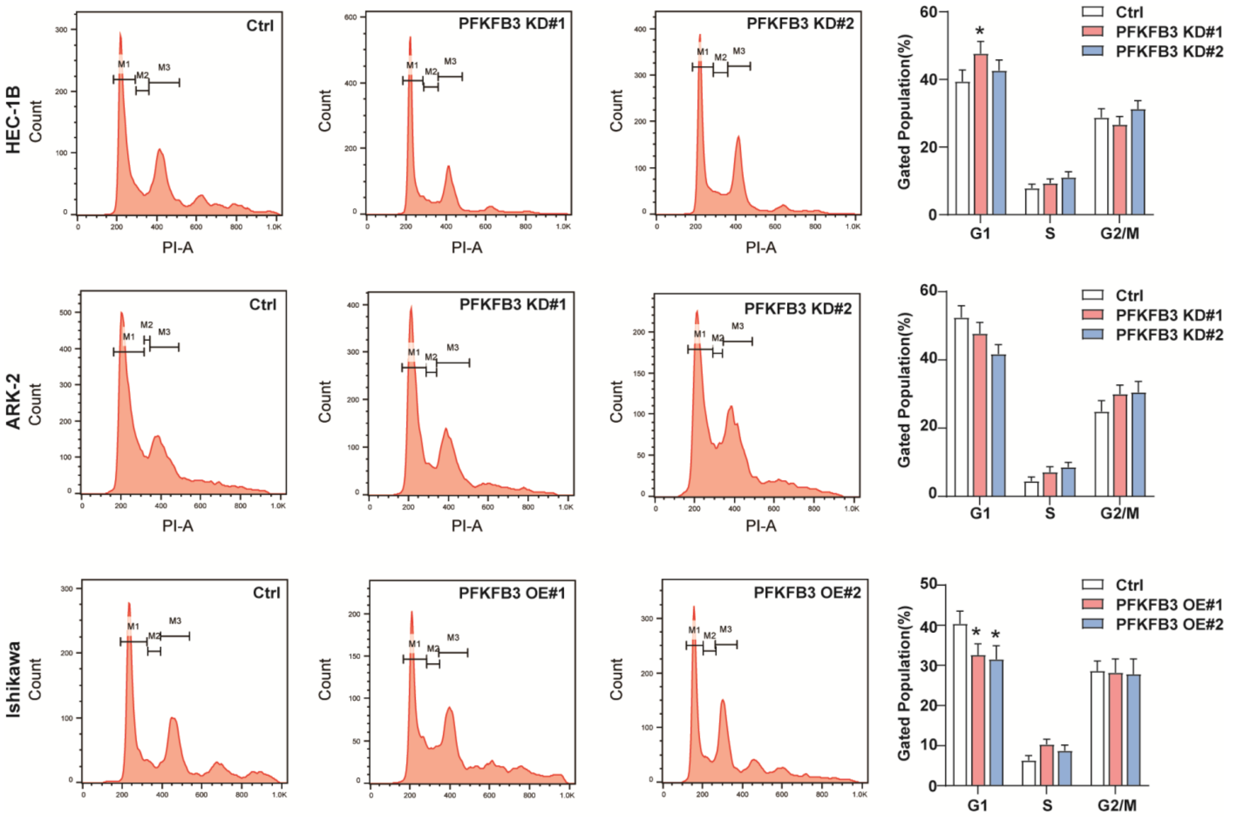

Fig. S7 Effects of PFKFB3 knockdown and overexpression on G1 cell cycle arrest in EC cells.

Cell cycle distribution was analyzed by flow cytometry after PFKFB3 knockdown (KD) in HEC-1B, ARK-2 cells and PFKFB3 overexpression (OE) in Ishikawa cell by CRISPR/Cas9. *, $p < 0.05$.

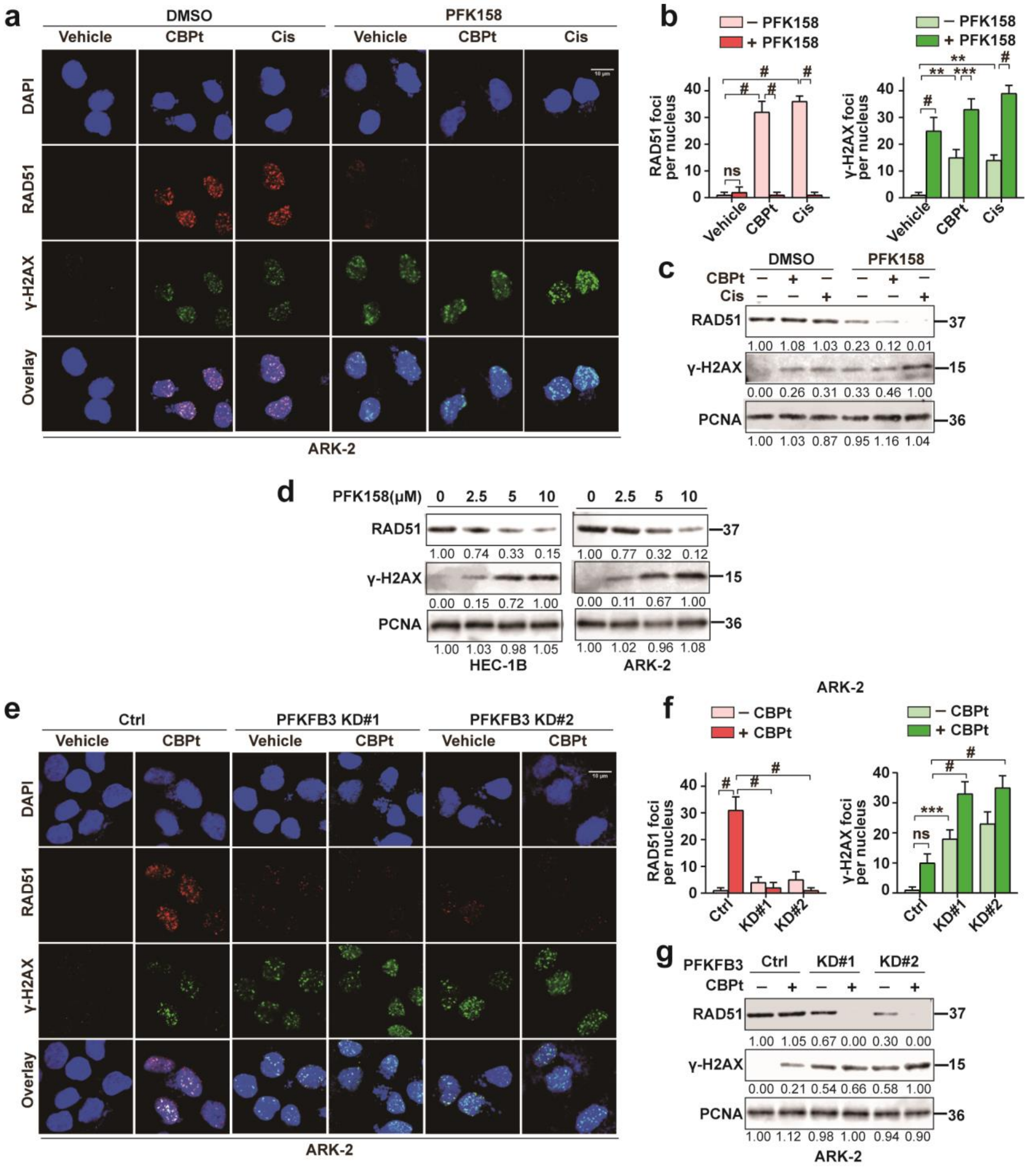

Fig. S8 (Related to Fig 6) PFKFB3 inhibition induces DNA damage and downregulates CBPt/Cis-induced RAD51 to disrupt DNA repair in EC cells

a Confocal analysis of RAD51 foci (red) and γ-H2AX foci (green) in ARK-2 cells, following treatment with CBPt (100μM)/Cis (10μM), PFK158 (5μM) or their combination for 24h. n=3 independent experiments. Scale bar, 10μm. **b** Bar chart showing RAD51 foci (left panel) and γ-H2AX foci (right panel) as quantified using CellProfiler, n >100 cells/treatment. The data represent as mean ± SD of three independent experiments, **p*<0.05; ***p*<0.01; ****p*<0.001; #*p*<0.0001; ns, not significant. **c** Western blotting was performed to determine the expression of RAD51 and γ-H2AX proteins in treated ARK-2 cells. PCNA was used as a loading control. **d** Dose-dependent effect of PFK158 on RAD51 and γ -H2AX protein expressions was determined in HEC-1B and ARK-2 cells treated with PFK158 (0, 2.5, 5, 10μM) for 24h. The experiments were also done in ARK-2 cells after treatment with or without CBPt (100μM) for 24h after PFKFB3 KD (**e-g**).

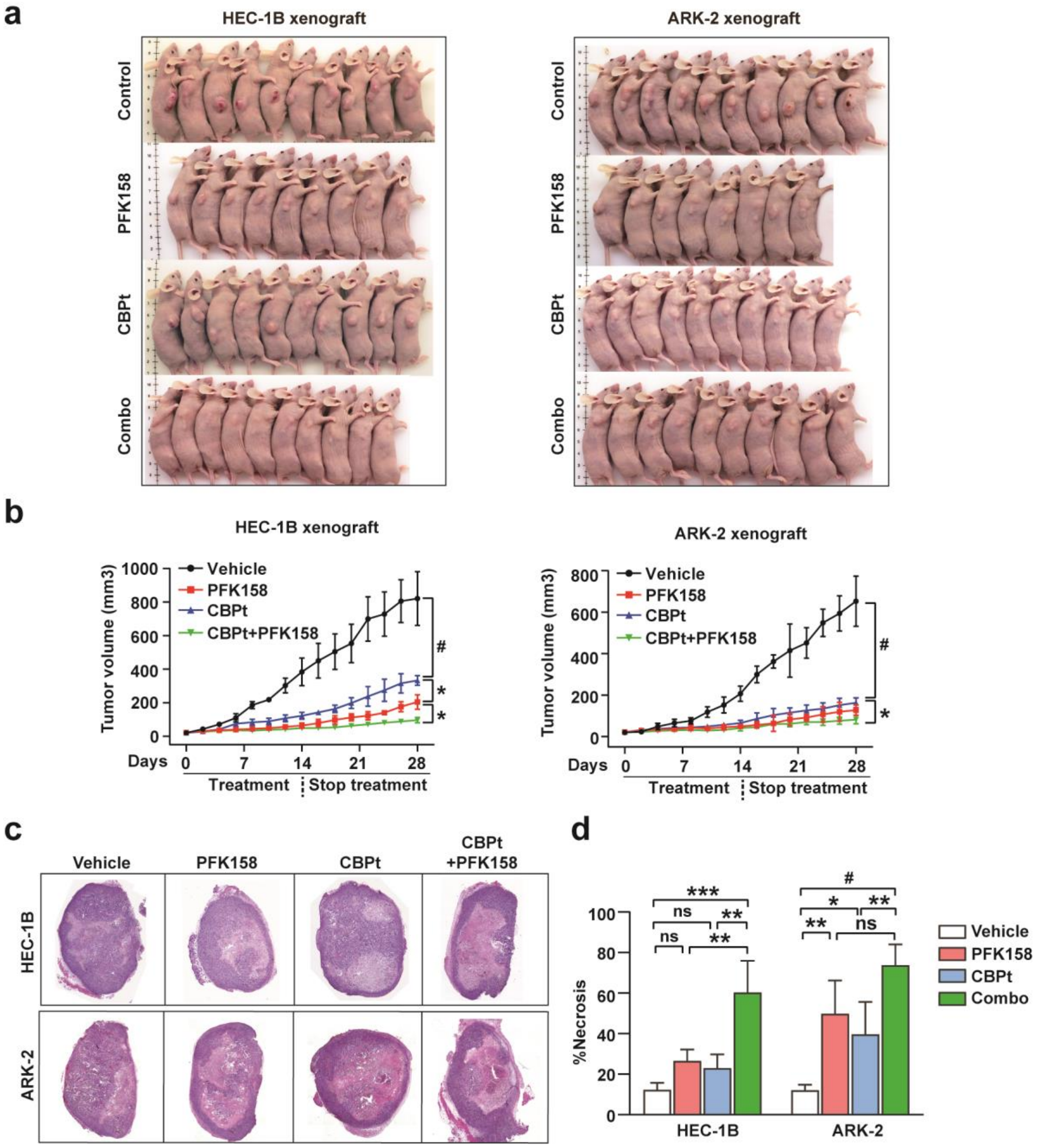

Fig. S9 (Related to Fig 7) Antitumor efficacy of PFK158 alone and in combination with carboplatin in two mouse xenograft models

a Typical photo of mice from different groups at the time of sacrifice are shown. **b** Effect of single-agent and dual treatment of PFK158 and CBPt on tumor growth of HEC-1B and ARK-2 cells in nude mice (n=8-10 per group). Tumor volume was determined every 2 days after the onset of treatment. **c** H&E staining of tumor sections for tumor histology after treatment. **d** Quantification of the necrotic area in mouse xenografts across treatment groups as demonstrated in H&E staining. Data are presented as mean ± SD (n=3, *$p < 0.05$, **$p < 0.01$, ***$p < 0.001$ and #$p < 0.0001$ for the indicated comparison; ns, not significant).
